# Neonatal Endogenous Endophthalmitis: A Case Report

**DOI:** 10.7759/cureus.22256

**Published:** 2022-02-15

**Authors:** Mustafa A Alhamoud, Ghadah H Alnosair, Hassan Y Alhashim

**Affiliations:** 1 Ophthalmology, King Fahd Hospital of the University, Dammam, SAU; 2 Pediatric Ophthalmology, Dammam Medical Complex, Dammam, SAU; 3 Ophthalmology, Imam Abdulrahman Bin Faisal University, Dammam, SAU

**Keywords:** intraocular infection, neonatal infection, ophthalmology, ocular infection, endogenous endophthalmitis

## Abstract

The aim of this study is to share our experience of a baby boy patient who presented with rare endogenous endophthalmitis that ended up with exudative retinal detachment; emphasizing the clinical presentation, follow-ups progression, and the management plan.

A case report of a one-month-old preterm baby boy presented with eye discharge in his left eye (OS) associated with eyelid swelling and chemosis for four days. His clinical examination revealed a congested left eye with proptosis, absent red reflex, and normal intraocular pressure (IOP) while a portable slit-lamp examination showed an edematous left eye with cloudy cornea but no infiltrates and no view to the posterior segment. Blood, cerebrospinal fluid (CSF), and ocular discharge were cultured, and all came negative and the patient started on empirical antibiotics. B-scan shows dense infiltrates in the vitreous cavity with subretinal fluid. Diagnostic intravitreal paracentesis was done which showed the growth of *Pseudomonas aeruginosa *and a diagnosis of endogenous endophthalmitis is made then a directed management plan was initiated. Unfortunately, a few days later a repeated B-scan was ordered to the left eye and it shows exudative retinal detachment, and a referral to retinal surgery service was consulted. After further follow-ups, B-scan showed resolving retinal detachment with a short shrunken eye, marked ocular wall thickening, and a relatively short axial length which is consistent with prephthisical changes hence, an oculoplasty referral was done for ocular prosthesis later on.

Endogenous endophthalmitis is a rarely encountered intraocular infection yet it carries devastating consequences that may threaten vision. Therefore, a high index of suspicion is essential for early detection of the disease to prevent serious complications and achieve good visual outcomes.

## Introduction

Endophthalmitis is an infectious condition of the internal ocular spaces, however clinically it is usually used to refer to inflammation secondary to intraocular infection. Endogenous bacterial endophthalmitis is a rare condition responsible for only 2% to 8% of endophthalmitis cases [1]. The rare occurrence of neonatal endogenous endophthalmitis is suggested by its incidence in the United States where In 1998, 317 babies were diagnosed with neonatal endogenous endophthalmitis out of 3.64 million newborns (8.71 cases per 100,000 live births). In 2006, only 183 neonates (4.42 cases per 100,000 live births) were diagnosed with neonatal endogenous endophthalmitis out of 4.14 million newborns. Throughout 1998 and 2006, the incidence of endophthalmitis declined at a rate of 6% per year (P =.01130) [2]. Despite the rare occurrence of endophthalmitis in general, it can lead to an aggressive intraocular infection that results in a poor visual prognosis.

Endophthalmitis is classified into endogenous and exogenous forms based on the route of infection. Exogenous endophthalmitis may be a result of penetrating eye injury, intraocular surgery, corneal ulcer, or periocular infection invasion of external barriers that protect the eye. The exogenous form of endophthalmitis is known to be more common than the endogenous causes. On the other hand, endogenous endophthalmitis also known as metastatic endophthalmitis results from organisms’ invasion of the intraocular space through the bloodstream after breaking the blood-ocular barrier [3].

## Case presentation

**Figure 1:**
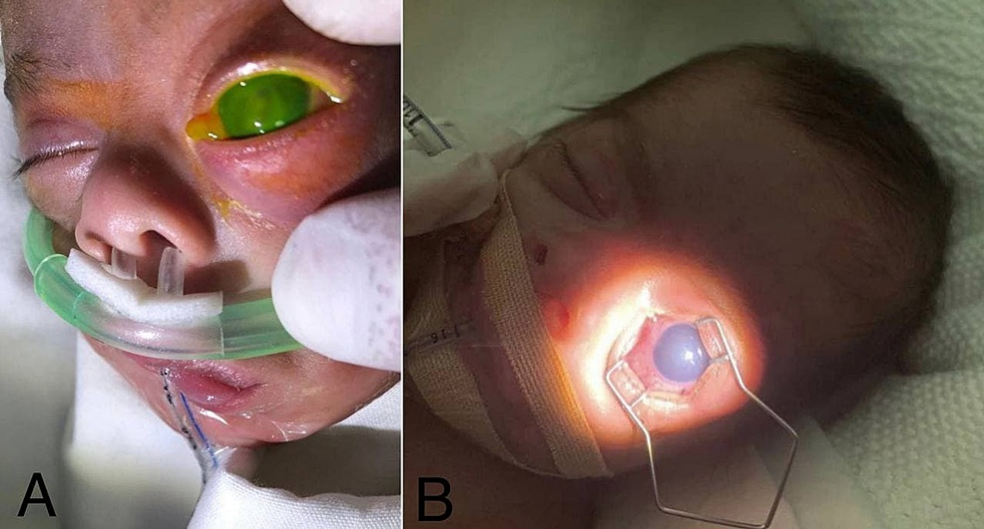
(A, B) External photograph of the left eye demonstrates periorbital edema causing proptosis, diffuse chemosis, hyperemia, corneal edema but no hypopyon filling the anterior chamber with absent red reflex.

We received an ophthalmology consultation to evaluate a one-month-old Saudi preterm baby boy to a mother with severe preeclampsia on a maximum dose of magnesium sulfate born via cesarean section due to severe fetal distress at 29 weeks + 3 days of gestational age. The perinatal period was complicated by intrauterine growth restriction (IUGR), a very low birth weight of 1 kg, respiratory distress syndrome (RDS), and necrotizing enterocolitis the patient was then intubated and sent to the neonatal intensive care unit (NICU). presented to our emergency department (ED) in Maternity and Children Hospital in Dammam, Saudi Arabia, complaining of an eye discharge in his left eye (OS) associated with eyelid swelling and chemosis for four days. His clinical examination revealed a congested left eye with proptosis, absent red reflex, and normal intraocular pressure (IOP) while a portable slit-lamp examination showed an edematous left eye with cloudy cornea but no infiltrates and no view to the posterior segment. His right eye (OD) examination is completely normal (Figures 1A, 1B). As a baseline assessment of the retina, RetCam imaging was ordered to exclude coexistent retinopathy of prematurity (ROP) which was normal.

Extensive laboratory and radiological workups were ordered previously upon the onset of symptoms by the primary team which includes a complete blood count (CBC) showing leukocytosis with neutrophil predominance. Blood, cerebrospinal fluid (CSF), and ocular discharge were cultured, and all came negative. Regarding the radiological assessment, orbital and cranial computed tomography (CT) scan with contrast was ordered and showed the left orbit appears mildly enlarged as compared to the right side with about 2 mm difference with left orbital proptosis, mild left orbital sclera thickening with mild enhancement is noted associated with heterogenous echogenicity within the vitreous, and mild preseptal soft-tissue thickening and enhancement is seen denoting preseptal cellulitis with no collection or intracranial extension.

**Figure 2:**
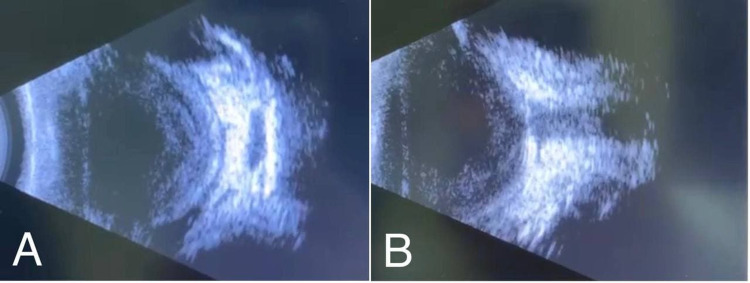
(A, B) B-scan ultrasound pictures of the left eye demonstrate dense infiltrates in the vitreous cavity mostly peripheral, choroid, and retina with subretinal fluid without evidence of shadowing.

**Figure 3:**
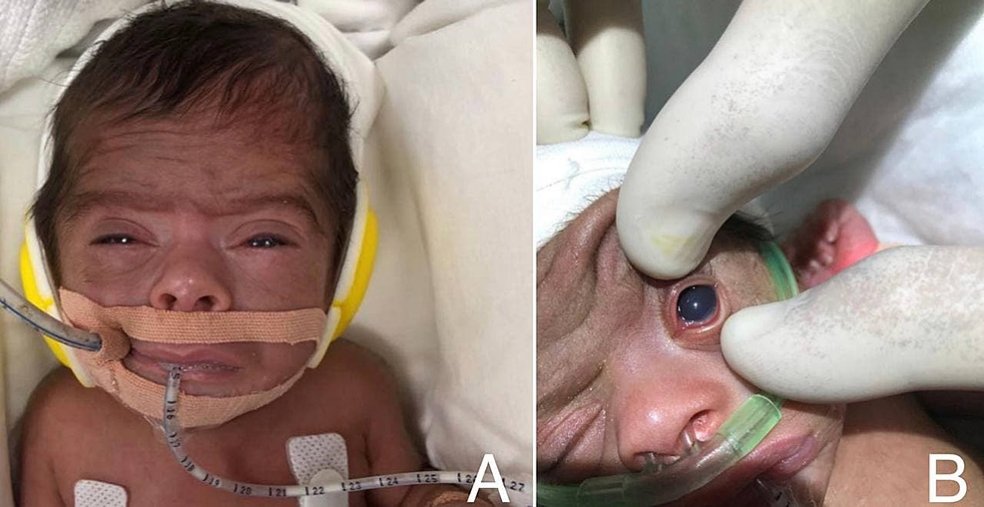
(A, B) External photograph of the left eye demonstrates a three-day post-injection improvement. The periocular edema, proptosis, and conjunctival chemosis were markedly improved, and corneal cloudiness decreased.

**Figure 4:**
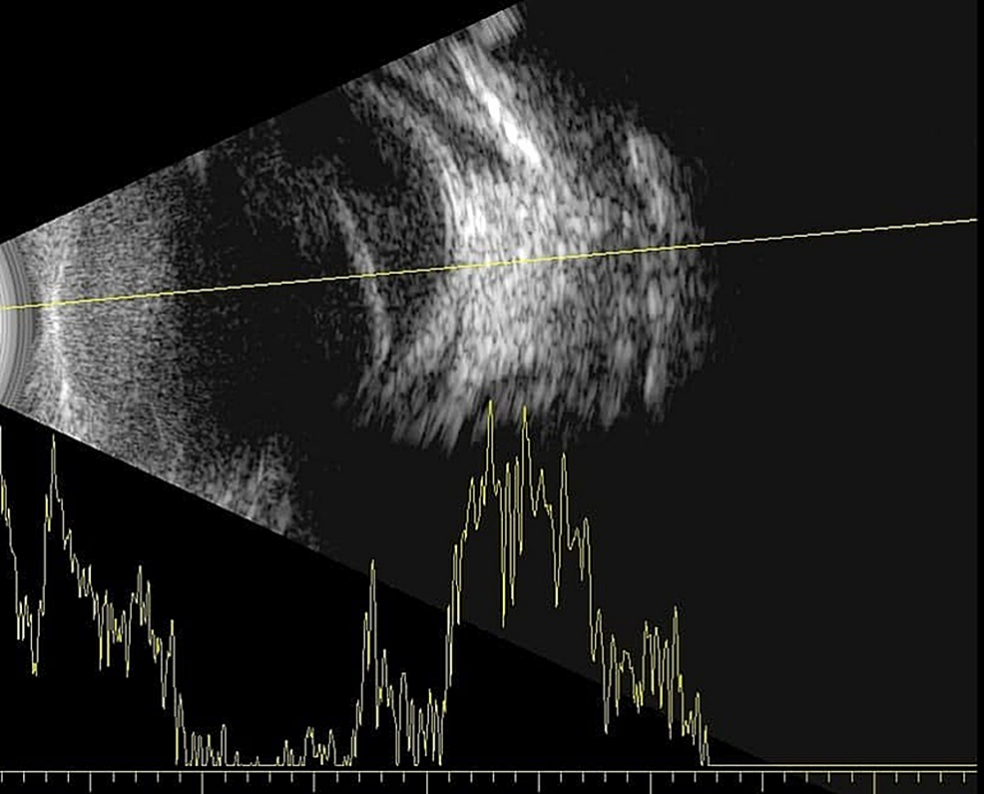
B-scan ultrasound picture of the left eye showing funnel-shape exudative retinal detachment.

The patient was started on empirical antibiotics including gentamycin eye drops for the left eye Q8h, amikacin 15 mg/kg IV, vancomycin 15 mg IV initiated by the primary team. Then, the infectious disease team was consulted to adjust the antibiotics according to the CT findings and they change the amikacin to tazocin 100 mg IV for better tissue penetration. After that, the ophthalmology consultation team carried out the case and we did an orbital ultrasound and B-scan shows dense infiltrates in the vitreous cavity with subretinal fluid (Figures 2A, 2B). We did a diagnostic intravitreal paracentesis to the left eye with the injection of empirical antibiotics simultaneously which include vancomycin and ceftazidime 1 mg/0.1 mL injection and dexamethasone 40 mg injection into the vitreous cavity using a 30-gauge needle 1 mm away from the limbus and the vitreous sample was sent for the culture and sensitivity. We measure the IOP post-injection which was 26 so Cosopt (Dorzolamide Hydrochloride-Timolol Maleate Ophthalmic Solution) was applied then subsequently the IOP became 16. 3 days post-injection the left eye became quiet and the proptosis improved, the cornea is clear and a remnant posterior synechia was observed, the anterior chamber was deep and sill no red reflex while the right eye examination was completely normal (Figures 3A, 3B). After one day of the procedure, while waiting for the result of the vitreous paracentesis culture, repeated urine culture was positive for *Pseudomonas aeruginosa* and the vitreous sample showed the growth of *P. aeruginosa* too therefore a diagnosis of endogenous endophthalmitis is established and we added moxifloxacin eye drops Q6 h, gentamycin eye drops Q8 h, gentamycin 4 mg/kg IV beside the same systemic antibiotics, and daily follow-ups were applied. Unfortunately, a few days later a repeated B-scan was ordered to the left eye and it shows funnel-shape exudative retinal detachment, and a referral to retinal surgery service was consulted (Figure 4).

**Figure 5:**
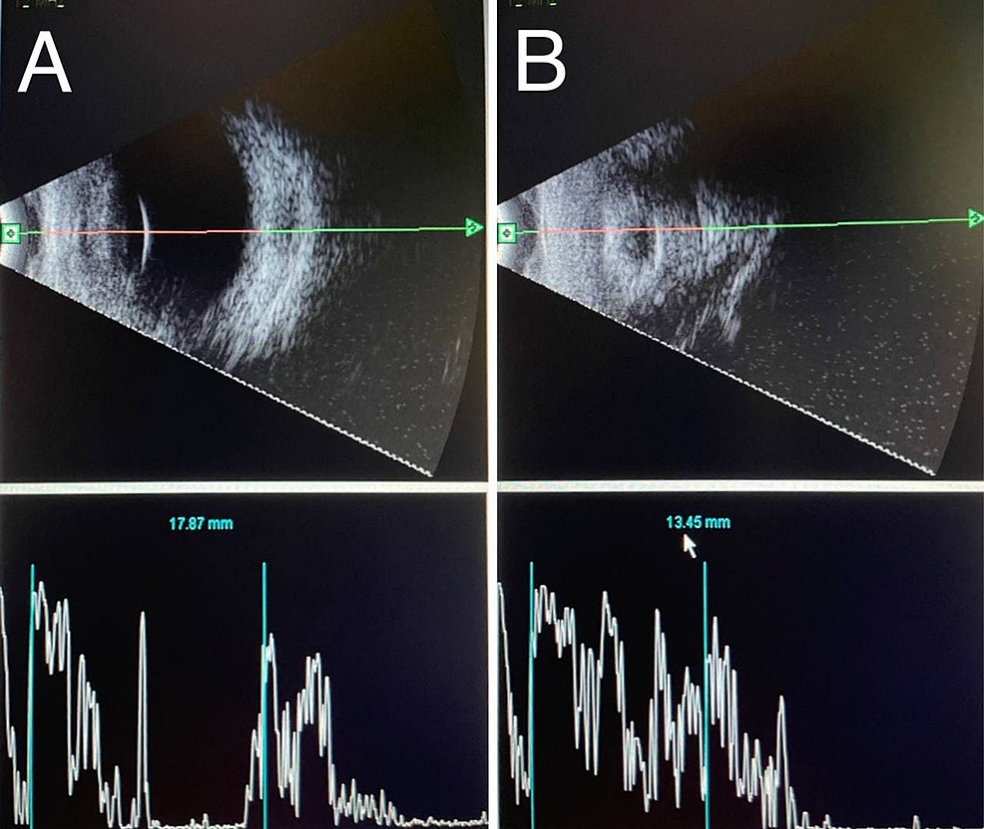
A repeated B-scan: (A) Right eye (OD): normal, the axial length is 17.78 mm. (B) Left eye (OS): shorth shrunken eye, marked ocular wall thickening, no retinal detachment and the axial length is 13.45 mm, which is consistent with prephthisical changes.

During multiple follow up later, clinical examination of the right eye (OD) was completely normal while the left eye (OS) started to regress and it becomes smaller in size with a clear cornea, 360° synechia, shallow anterior segment, multiple fibrous retrolental membranes with cataract, and no view to the fundus. Therefore, a repeated B-scan was planned, which revealed a normal examination of the right eye (OD) with an axial length of 17.87 mm while the left eye (OS) showed resolving retinal detachment with a short shrunken eye, marked ocular wall thickening and the axial length was 13.45 mm, which is consistent with prephthisical changes; hence, vitreoretinal team’s opinion was against any surgical intervention and a referral for oculoplastic clinic was done for ocular prosthesis later on (Figures 5A, 5B).

## Discussion

Endophthalmitis is an infectious condition of the internal ocular spaces. Despite its rare occurrence in general. it carries an aggressive course of disease that may result in a poor visual outcome.

The causative organisms of endogenous endophthalmitis might be bacterial or fungal sources. For bacterial causes, the most common causative organisms are divided between gram-negative and gram-positive. For gram-negative, the most frequent organisms are *Klebsiella pneumonia*, *P. aeruginosa*, and *Neisseria meningitides*. On the other hand, the gram-positive bacteria are *Staphylococcus aureus*, group B Streptococci, *Streptococcus pneumoniae*, and *Nocardia* species were most commonly identified [4]. For fungal organisms, *Candida albicans* and *Aspergillus* are the most common organisms [5].

Endogenous endophthalmitis is rarely reported in healthy individuals as immunocompromised people are more susceptible to this type of infectious condition such as patients with diabetes mellitus, organ transplants, liver and kidney disease, cancer, and a patient who receive long-term steroid therapy [6]. In our case, the patient was admitted to the NICU and intubated after birth where contamination of indwelling lines and tubes is not an uncommon thing and is considered a potential source of septicemia and consequently neonatal endogenous endophthalmitis. Moreover, our case was a preterm baby who is underweight which together decreases the immunity and predispose to infections. Studies have shown that the majority of cases are the result of endogenous endophthalmitis transferred perinatally or during extended postnatal hospitalization [7].

Al-Khersan et al. reported that clinical examination of patients affected with endogenous bacterial endophthalmitis showed an injection of the affected eye, defect of the corneal epithelium, infiltrate, depression of the anterior chamber, obstructed view of iris and the posterior chamber, pus collection in the anterior chamber, and blood collection in the anterior chamber [8]. While Jackson et al. reported clinical features of affected patients which include decreased visual acuity, discomfort in the diseased eye, obstructed view of the fundus, pus collection, pyrexia, flu symptoms, and inflammation of the vitreous cavity and the anterior chamber. He also illustrated that in most cases, the systemic clinical features usually present before the onset of ocular manifestations [3]. Moreover, Vaziri et al. showed in their report that clinical presentation typically includes uveitis, poor vision, floaters, redness, eye pain, and photophobia. He also reported that the patient could be asymptomatic, and the condition usually affects one eye even though the bilaterality of the disease was reported in some studies [9]. However, our patient presented with left eye discharge, eyelid swelling, and chemosis. Upon clinical examination of the left eye, there was congestion, proptosis, undetectable red reflex, with normal IOP. When the portable slit-lamb examination was done it showed edema of the affected eye, corneal cloudiness, no infiltrate, and normal fundus.

Biranaum et al. stated that diagnostic workup for suspected endogenous endophthalmitis includes ocular fluid examination, blood tests, and imaging studies. For ocular fluid, gram stain, culture, and polymerase chain reaction (PCR) are usually performed. Furthermore, blood tests like CBC and liver function test, bacterial culture, and fungal culture are helpful in the diagnostic workup. Also, urine culture and CSF culture are tests that should be done as needed. Imaging studies including B-scan ultrasound, CT, and transesophageal echocardiogram play a vital role in the diagnosis [10]. While Jackson et al. reported that systemic laboratory workup together with clinical features is necessary to establish the diagnosis. Investigations such as CBC and blood culture are the most valid way to establish the diagnosis of endogenous bacterial endophthalmitis. He also reported the use of PCR recently to diagnose and identify the causative organisms. Blood culture in his study revealed that the most common organisms are *K. pneumonia*, *P. aeruginosa*, *N. meningitid*es, *S. aureus*, group B S*treptococcus*, and *S. pneumoniae* [4]. CBC test in our case showed increased leukocytes counts. while blood culture was negative together with CSF, and ocular discharge.

Moreover, Al-Khersan et al. reported in their study that blood culture was positive in some of the patients, however, in most of the patients, the diagnosis was made by culture of the aqueous or vitreous sample as in our case where *P. aeruginosa* was identified in the vitreous fluid after vitreous paracentesis. And in some cases, the diagnosis was made based on extraocular cultures most commonly urine culture as in our case where *P. aeruginosa* was initially identified. He also reported that the B-scan of the affected patient showed thick opacity in the vitreous without posterior shadowing in addition to the subretinal fluid collection which is similar to the findings in our case [8].

Generally, in terms of treatment, wide-spectrum antibiotics such as fluoroquinolones, aminoglycosides, third-generation cephalosporin, and clindamycin are the main agents. García-Sáenz et al. showed that the selection of appropriate antibiotics should be based on how broad the coverage and the ability of the antibiotics to penetrate the blood-ocular barrier. They also reported that even if the patient received intravitreal antibiotics the patient must receive systemic antibiotics also [11].

Smith et al. stated that systemic fluoroquinolones are excellent in passing the blood-ocular barrier, especially with repeated administration [12]. Ahmed et al. reported that for gram-negative coverage including pseudomonas, ceftazidime which is third-generation cephalosporin is used; however, it has limited ocular penetration in case of pseudomonas infection in comparison to other bacteria such as *Haemophilus influenzae*. He also illustrated that vancomycin when administered through the intravenous route has limited penetration ability to the blood-ocular barrier [13].

Binder et al. showed in their study that all the patients included in the study received intravenous and intravitreal antibiotics, on the other hand, a small number of patients received para-plana vitrectomy in combination with the antibiotics which didn’t result in better visual acuity [6]. While Jackson et al. reported that the patients included in his study were divided into three groups: one group was given systemic antibiotics in combination with intravitreal antibiotics, another group received only systemic antibiotics, and the third group received a combination of systemic and intravitreal antibiotics together with pars-plana vitrectomy. He illustrated that ceftazidime was mostly used for gram-negative coverage and vancomycin for gram-positive [4]. In another paper, Jackson et al. reported the use of vitreous biopsy and intravitreal administration of antibiotics simultaneously serves as a diagnostic and therapeutic procedure [3]. In our case, after the identification of *P. aeruginosa* in urine culture, the patient was given a number of antimicrobial agents empirically those agents including gentamicin, tazobactam, moxifloxacin, and fluconazole. After that intravitreal paracentesis was performed with injection of vancomycin, ceftazidime, and lastly dexamethasone as it helps in decreasing the inflammation which is also supported by other studies [14,15].

## Conclusions

Endogenous endophthalmitis is an uncommon intraocular infection usually of an unknown source yet it carries a substantial risk for visual deterioration. A high index of suspicions is needed for reaching an appropriate differential diagnosis which necessitates a prompt comprehensive investigation for early detection of the disease. Achieving directed therapy is essential to attain a good visual outcome via vision-saving interventions through an interdisciplinary approach. Addressing the potential risk factors for developing endogenous endophthalmitis should be the main aim for the prevention of such devastating infection.
